# Ex vivo Retrieval of Mature Oocytes for Fertility Preservation in a Patient with Bilateral Borderline Ovarian Tumor

**DOI:** 10.1055/s-0040-1718436

**Published:** 2021-01-19

**Authors:** Bruno Ramalho de Carvalho, Geórgia Fontes Cintra, Taise Moura Franceschi, Íris de Oliveira Cabral, Leandro Santos de Araújo Resende, Brenda Pires Gumz, Thiago David Alves Pinto

**Affiliations:** 1 Hospital Sírio-Libanês, Brasília, DF, Brazil; 2 Genesis, Centro de Assistência em Reprodução Humana, Brasília, DF, Brazil; 3 Diagnose, Laboratório de Anatomia Patológica e Citologia, Brasília, DF, Brazil

**Keywords:** cryopreservation, ex vivo oocyte retrieval, fertility preservation, ovarian cancer, borderline ovarian tumor, vitrification, criopreservação, recuperação extracorpórea de oócitos, preservação de fertilidade, câncer do ovário, tumor borderline de ovário, vitrificação

## Abstract

We report a case of ultrasound-guided ex vivo oocyte retrieval for fertility preservation in a woman with bilateral borderline ovarian tumor, for whom conventional transvaginal oocyte retrieval was deemed unsafe because of the increased risk of malignant cell spillage. Ovarian stimulation with gonadotropins was performed. Surgery was scheduled according to the ovarian response to exogenous gonadotropic stimulation; oophorectomized specimens were obtained by laparoscopy, and oocyte retrieval was performed ∼ 37 hours after the ovulatory trigger. The sum of 20 ovarian follicles were aspirated, and 16 oocytes were obtained. We performed vitrification of 12 metaphase II oocytes and 3 oocytes matured in vitro. Our result emphasizes the viability of ex vivo mature oocyte retrieval after controlled ovarian stimulation for those with high risk of malignant dissemination by conventional approach.

## Resumo

Relatamos um caso de obtenção ex vivo de óvulos, guiada por ultrassonografia, para preservação da fertilidade em uma mulher com tumor ovariano borderline bilateral, para quem a recuperação transvaginal convencional foi considerada insegura, devido ao aumento do risco de disseminação de células malignas. Foi realizada estimulação ovariana com gonadotrofinas. A cirurgia foi agendada de acordo com a resposta ovariana à estimulação gonadotrófica exógena; após ooforectomia por laparoscopia, ∼ 37 horas após a maturação folicular, procedeu-se à recuperação extracorpórea de oócitos. Um total de 20 folículos ovarianos foi aspirado e 16 complexos cumulus foram obtidos, resultando na vitrificação de 12 oócitos maduros e de 3 oócitos imaturos amadurecidos in vitro. Nosso resultado enfatiza a viabilidade da recuperação ex vivo de oócitos maduros após estimulação ovariana controlada para mulheres com alto risco de disseminação maligna pela captação oocitária realizada convencionalmente pela via transvaginal.

## Introduction

According to the literature, the incidence of borderline ovarian tumors (BOTs) in women < 45 years old varies between 27 and 59%. 1 2 3 Indeed, the expected good prognosis of BOT and the current trends of having a first baby after 30 years old lead to the fact that more women presenting with the tumor should not have started their offspring yet at the time of diagnosis. Moreover, childbearing has been reported to be an important issue for BOT survivors. 3 Then, the issue of both conservative approach of ovarian tumors and alternative options to preserve childbearing potential becomes definitely important.

In the context, fertility preservation strategies arise with the aim of improving and maintaining the quality of life after cancer. Fertility-sparing surgery is a viable alternative for young women presenting BOT, since they have better prognosis when compared with other malignancies of the female gonad. 4 However, oocytes are usually obtained by endovaginal puncture of the ovaries and the procedure is considered to be unsafe in the presence of adnexal tumors, given the risk of tumor capsule rupture and malignant cells spillage.

The feasibility of ex vivo egg collection has been demonstrated and it has been a seemingly successful strategy, according to case reports and series, especially involving the aspiration of immature eggs, then submitted to in vitro maturation (IVM) before vitrification. 5 6 7 8 9 10 As a matter of fact, in spite of being a patient-friendly intervention, reducing costs and avoiding the risk of ovarian hyperstimulation syndrome, the outcomes of IVM are not sufficiently good for its use as a technique of choice in assisted reproduction centers all over the world. Moreover, information on risks is still lacking, especially those related to genetic and epigenetic alterations. 11

In a scenario where the reproductive outcomes from oocytes matured in vitro seem to be worse than those obtained from in vivo mature oocytes, 11 ovarian stimulation for the ex vivo retrieval of mature eggs has been eventually performed and documented as a possible strategy. 12 13 14 15 In the present paper, we report a case of ultrasound-guided retrieval of mature oocytes from stimulated ovaries after laparoscopic bilateral salpingo-oophorectomy, in a woman with bilateral borderline serous ovarian tumor.

## Case Report

A 28-year-old married nulligravida, weighting 62.5 kg (body mass index 21.37 kg/m ^2^ ), was referred by her gynecologic oncologist on July 2019 for an emergency consultation regarding fertility preservation options, due to the finding of a bilateral adnexal mass, with the suspicion of being a borderline tumor. Indeed, there was an elevated chance of malignancy according to the criteria stablished by the International Ovarian Tumor Analysis group, 16 based on the finding of an irregular solid tumor with capsular projections in the right ovary, and elevated tumor marker CA-125. Bilateral salpingo-oophorectomy was the proposed treatment.

Five days after the initial consultation (day 14 of the menstrual cycle), the patient received corifollitropin alfa (Elonva, Schering-Plough, Kenilworth, NJ, USA) in a subcutaneous (SC) single dose of 150 µg, for controlled ovarian stimulation (COS). Endovaginal ultrasound allowed the identification of 15 and 12 antral follicles < 10 mm in the right and left ovaries, respectively, and no dominant follicles were observed. To prevent premature Luteinizing Hormone (LH) surge, SC daily single doses of the GnRH antagonist ganirelix acetate (Orgalutran, Schering-Plough, Kenilworth, NJ, USA) were administered from day 6 onwards. From treatment day 8 to day 10, ovarian stimulation was continued by SC daily doses of 250 IU of recombinant follitropin (Puregon, Schering-Plough, Kenilworth, NJ, USA). A SC dose of 0.2 mg of the gonadotropin-releasing hormone agonist triporelin acetate (Gonapeptyl Daily, Ferring, Saint-Prex, Switzerland) was administered for final follicular maturation on treatment day 11, when at least 11 follicles were expected to present with a mean diameter ≥ 16 mm.

Surgery was scheduled according to the ovarian response to gonadotropic stimulation, and the patient was conducted to the operating room 36 hours after the ovulatory trigger. Bilateral salpingo-oophorectomy was proceeded by laparoscopy and both gonads were placed in plastic endobags for cavity protection, following the oncological procedure to avoid the dissemination of the disease. Extraction, then, was performed through a 5 cm vertical midline incision in the abdominal wall. The endobags containing the removed nonruptured ovaries were taken to the oocyte ex vivo retrieval set by the main surgeon, in the operating room, with a maximum ischemia time of 6 minutes.

**Fig. 1:**
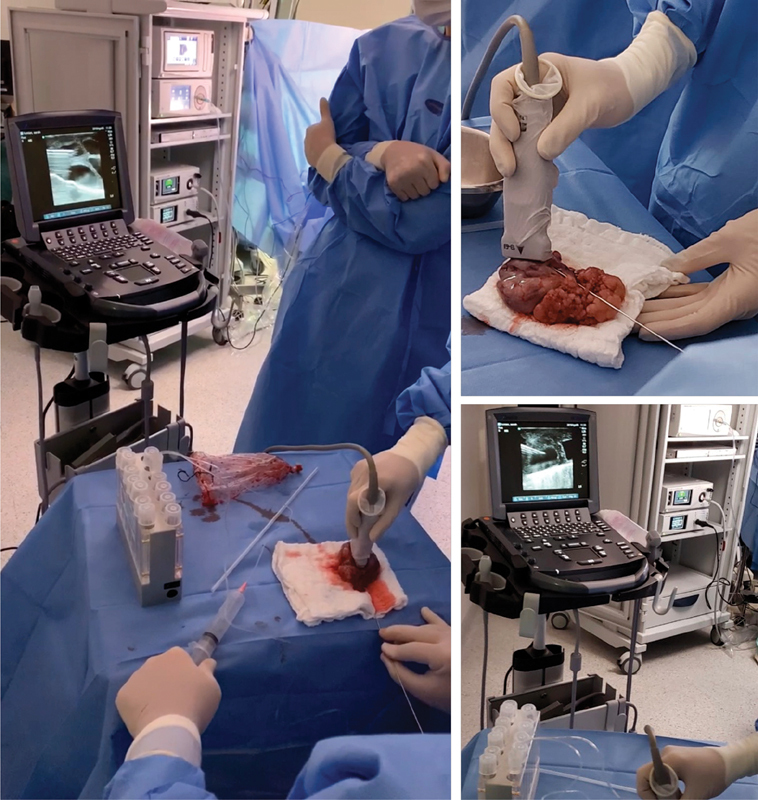
Oocyte retrieval set. Up in the right, follicular aspiration with a standard aspiration single lumen needle, in a closed system connected directly to the tubes, under ultrasound guidance, using a 6–13 MHz linear probe applied directly to the specimen. Down in the right, sonographic view of the needle during guided follicular aspiration.

In the oocyte retrieval set, the ovaries were placed over a sterile surgical cloth at room temperature. Oocyte retrieval was performed ∼ 37 hours after the ovulatory trigger, with a standard aspiration single lumen needle (Wallace 17G Oocyte Recovery Set 330 mm; Smiths Medical International, UK), in a closed system connected directly to the tubes, which were placed in a preheated tube warmer (model TW37, Origio, Målov, Denmark). All enlarged follicles were punctured and aspirated under ultrasound guidance, using a 6–13 MHz linear probe applied directly to the specimens ( Fig. 1 ).

A total of 20 ovarian follicles were aspirated, filling ten 14 mL tubes with follicular fluids, which were transported in the tube warmer, placed in a thermal box, with the intention of simulating optimal temperature to the embryology laboratory. The journey from the hospital to the laboratory was of ∼ 9 km, completed in ∼ 16 minutes.

**Fig. 2:**
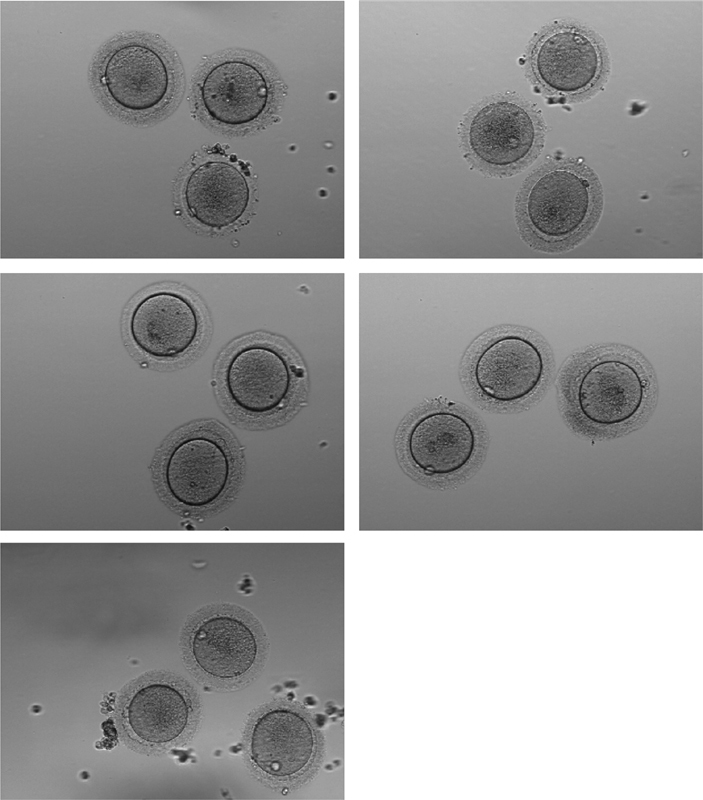
Photomicrograph, 200x, of the mature oocytes which were vitrified.

Sixteen *cumulus* complexes were obtained. *Cumulus* cells were removed with hyaluronidase 40 IU/mL, yielding 12 metaphase II (mature) and 3 metaphase I (immature) oocytes, and 1 germinal vesicle. The 3 immature oocytes were matured in vitro, and the final 15 mature oocytes obtained ( Fig. 2 ) were vitrified, 2,5 hours after being yielded, using a modified oocyte vitrification method as previously described by Kuwayama. 17

**Fig. 3:**
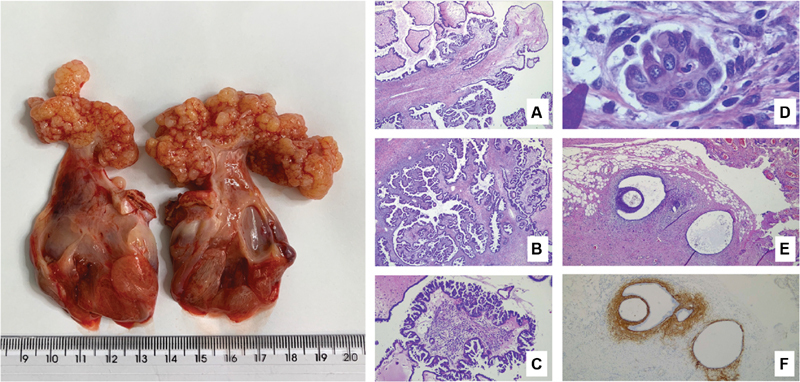
Macroscopic external view of the tumor in both ovaries. Photomicrographs, in hematoxycilin-eosin, are disposed as: ( **A** ) 40x, branched fibroconjunctive papillae covered by serous epithelium, sometimes forming micropapillae. ( **B** ) 40x, area of endophytic growth, with no evidence of stromal invasion. ( **C** ) 100x, area with micropapillae, measuring < 5.0 mm (which means that it is not a low-grade noninvasive serous carcinoma). ( **D** ) 800x, microinvasion focus in stroma, < 5.0 mm, consisting of terminally differentiated/senescent-looking epithelioid cells (no clinical repercussions). (E) 40x, focus of peritoneal endometriosis containing glands and stroma. (F) Immunohistochemistry, CD 10 positive in stromal cells of the endometriosis focus.

Of note, the entire procedure was performed using absolute sterile steps. The surgical staging procedure was completed by oncological surgeons, and after safety evaluation, the uterus was preserved. Final histopathology confirmed the diagnosis of bilateral serous borderline tumor on the surface of both ovaries with microinvasion of 0.2 mm ( Fig. 3 ), without tubal commitment. As an accidental finding, endometriosis implants were identified in the rectosigmoid and in the right uterosacral ligament, also confirmed by histopathology. Finally, the postoperative follow-up of CA-125 levels showed a decrease from 273 U/mL in day 10 after surgery to 9.62 U/mL after 3 months, and 7.09 U/mL in the last mensuration, which was made 4 days before we finished this report.

## Discussion

We report a case of ultrasound-guided ex vivo retrieval of mature oocytes for fertility preservation in a woman with bilateral borderline ovarian tumor, for whom conventional transvaginal follicular aspiration was deemed unsafe because of the increased risk of malignant cell spillage. To our knowledge, this is the first report of an ex vivo retrieval of mature oocytes in Brazil, and the second using standard ultrasound guidance in the international literature.

Conservative surgery with the intention of preserving the ability to conceive has been increasingly practiced in surgical gynecological oncology and is no longer limited to ovarian transposition before radiotherapy. Preservation of the pelvic anatomy is currently desirable for young women without constituted offspring, especially concerning the uterus, and, in many cases, at least the contralateral ovary. 18 19 In the meantime, there is consensus on the recommendation of caution regarding conservative surgery for hormone-sensitive ovarian tumors, as well as for high-risk serous borderline tumors 20 and those in advanced stages. 3

To date, the safety of fertility sparing approaches to different ovarian tumors in childbearing age patients really seems to be realistic. The recent meta-analysis of eight observational studies, comparing 2,223 women undergoing conservative surgery with 5,809 undergoing radical surgery did not find differences in overall survival and disease-free survival with either surgical techniques for stage 1 epithelial ovarian cancer. 21 In the same way, observational studies that evaluated the prognosis of malignant ovarian germ cell tumors 22 suggested that fertility sparing surgery is a safe treatment option, regardless of tumor stage and histological type, and a recent encouraging French casuistic demonstrated the experience of live births in two thirds of the women submitted to conservative surgery for borderline ovarian tumors, some occurring after recurrence. 23

As interventions to be added to the oncological approach with the aim to preserve female reproductive capacity, techniques of cryopreservation of oocytes, embryos, or even the ovarian cortex have been increasingly used worldwide. 24 25 26 27 In oncological cases, cryopreservation of mature oocytes seems to be the most interesting option, mainly for single women, those who do not wish to use donor sperm, or have religious or ethical objections to embryo freezing. 26 28 Fortunately, pregnancy rates from in vitro fertilization of frozen eggs are very close to those seen with fresh eggs nowadays. 29

It is noteworthy, however, that oocyte recovery is conventionally performed by follicular puncture through the vaginal fornix and, therefore, carrying the risk of rupture of the tumor capsule and tumor cells spillage, then changing the prognosis of the disease. Ex vivo recovery of oocytes eliminates that risk of peritoneal dissemination and the literature on the subject has been built from reports and case series. Conversely, oftentimes the puncture of oophorectomized specimens is proceeded without any stimulation, obtaining immature oocytes to be subsequently matured in vitro. 5 7 8 10 As an example, in the series of 34 cases published by Segers et al, 9 ex vivo recovery of immature oocytes resulted in obtaining mature oocytes in vitro or embryos available for cryopreservation in 79% of women, with an overall maturation rate of 36% after IVM.

In spite of being a widely studied technique, IVM has not reached the status of a routine or widely used technique, since outcomes are far below expectations. 11 For this reason, developing protocols and techniques that allow the capture of in vivo matured oocytes means a great advance for fertility preservation in women with ovarian malignancies.

To date, there are four case reports of ex vivo retrieval of mature oocytes after ovarian stimulation by exogenous gonadotropins in the literature. 12 13 14 15 Fatemi et al 12 and Bocca et al 13 were pioneers in the idea, both reporting the intervention occurring in women < 30 years old, and obtaining satisfactory amounts of mature oocytes, even aspirating the follicles identified by the external view. However, Pereira et al 14 recognized a technical limitation of the extracorporeal uptake of mature oocytes without ultrasound guidance, and the lack of this approach may be the reason for the small amount of gametes recovered, even though this was similar or superior to the number of oocytes obtained after ex vivo recovery of immature oocytes in published studies that included young women. 8 10

De la Blanca et al 15 were the first to use ultrasound guidance directly applied to the specimens; an endovaginal probe was used to facilitate access to the follicles. Unfortunately, the number of mature eggs retrieved was not exactly satisfactory, especially considering that the patient was 31 years old and the ovarian reserve seemed to be normal. The reports describing the preservation of mature oocytes obtained by ex vivo capture are summarized in Table 1 .

**Table 1:** Summary of fertility preservation case reports using ex vivo retrieval of mature oocytes in ovarian tumors

Reference	Age	Marital status; parity; brief medical history	COS	Surgery type	Pathology	US guidance (yes/no)	Total mature oocytes yielded	Total in vitro matured oocytes
Fatemi et al (2011) 12	27	Not mentioned; nulliparous; previous infertility reported; previous laparoscopic left salpingo-oophorectomy, papillary serous adenocarcinoma; ovarian reserve not mentioned	rFSH 200 IU/day; ganirelix acetate, 0,25 mg/day, from day 6; maturation trigger with urinary hCG 10,000 IU	Laparotomy	Papillary serous adenocarcinoma (recurrence)	No	13 ^†^	0
Bocca et al (2011) 13	25	Single; nulliparous; previous laparoscopic left salpingo-oophorectomy, serous borderline ovarian tumor; AFC ∼ 10	rFSH 200 IU/day; ganirelix acetate, 0,25 mg/day, from day 7 to day 10; maturation trigger with rhCG 250 µg on day 10	Laparoscopy, ∼ 34–35 hours after maturation trigger ^‡^	Serous borderline tumor	No	14	0
Pereira et al (2017) 14	37	Single; nulliparous; AFC ∼ 14	rFSH 300 IU/day + hpHMG 150 IU/day + letrozol 5 mg/day; rFSH reduced to 150 IU/day from day 8 to day 11; ganirelix acetate, 0,25 mg/day; maturation trigger with rhCG 250 µg on day 12	Laparotomy, ∼ 34 hours after maturation trigger	Not mentioned	No	7	0
de la Blanca et al (2018) 15	31	Single; nulliparous; previous laparoscopic left salpingo-oophorectomy, mature teratoma; AFC unfeasible, AMH 1.1 ng/mL	Chorifollitropin α 150 µg, rFSH 200 IU/day from day 8 to day 9; ganirelix acetate, 0,25 mg/day, from day 6 to day 10; maturation trigger with rhCG 250 µg on day 10	Laparoscopy, ∼ 35 hours after maturation trigger	*Struma ovarii*	Yes	5	0

Abbreviations: AFC, Antral Follicle Count; AMH, Anti-Müllerian Hormone; COS, Controlled Ovarian hyperStimulation; hCG, Human Chorionic Gonadotropin; hpHMG, highly purified Menotropin; rhCG, recombinant Human Chorionic Gonadotropin; rFSH, recombinant Follicle Stimulating Hormone.

† Intracytoplasmic sperm injection was proceeded, and 7 top quality zygotes were vitrified.

‡ Information obtained with the main author, Silvina Bocca, by e-mail, on May 6, 2020.

We believe that the use of a 6–13 MHz linear probe for ultrasound guidance to ovarian follicles aspiration helped us to achieve what we considered an excellent number of vitrified mature eggs. Also, it seems that we are the first to use IVM as an additional intervention, resulting in the highest number of mature vitrified oocytes for women with BOT ever published.

Of note, ovarian stimulation has not been associated with recurrence of ovarian malignancies to date, 30 even in women with a high-risk Breast Cancer Gene (BRCA) mutation. 31 Moreover, reports of live births following in vitro fertilization (IVF) after fertility-sparing surgery in patients with ovarian tumors suggest that pregnancy outcomes may be even better for them than those observed for infertile women, and that assisted reproductive techniques have no negative impact on the prognosis of cancer. 32

## Conclusion

In conclusion, our report emphasizes the viability of ex vivo mature oocyte retrieval after controlled ovarian stimulation for those with high risk of malignant dissemination by the conventional vaginal approach. Also, it reinforces the benefit of using ultrasound guidance for the access to ovarian follicles, which can be an important additive to achieve the best possible result.
